# Supplementary material for: Restricted differentiative capacity of Wt1-expressing peritoneal mesothelium in postnatal and adult mice
Source: Sci Rep. 2021 Aug 5;11:15940. doi: 10.1038/s41598-021-95380-1 (PMC8342433; doi:10.1038/s41598-021-95380-1)
Supplement: Supplementary file 1 — Supplementary Figures. [file 41598_2021_95380_MOESM1_ESM.docx]

Supplementary Figures to

**Restricted differentiative capacity of Wt1-expressing peritoneal mesothelium in postnatal and adult mice**

Thomas P Wilm^1^, Helen Tanton^1,3^, Fiona Mutter^1,4^, Veronica Foisor^1,5^, Ben Middlehurst^1^, Kelly Ward^1^, Tarek Benameur^1,6^, Nicholas Hastie^2^, Bettina Wilm^1^^


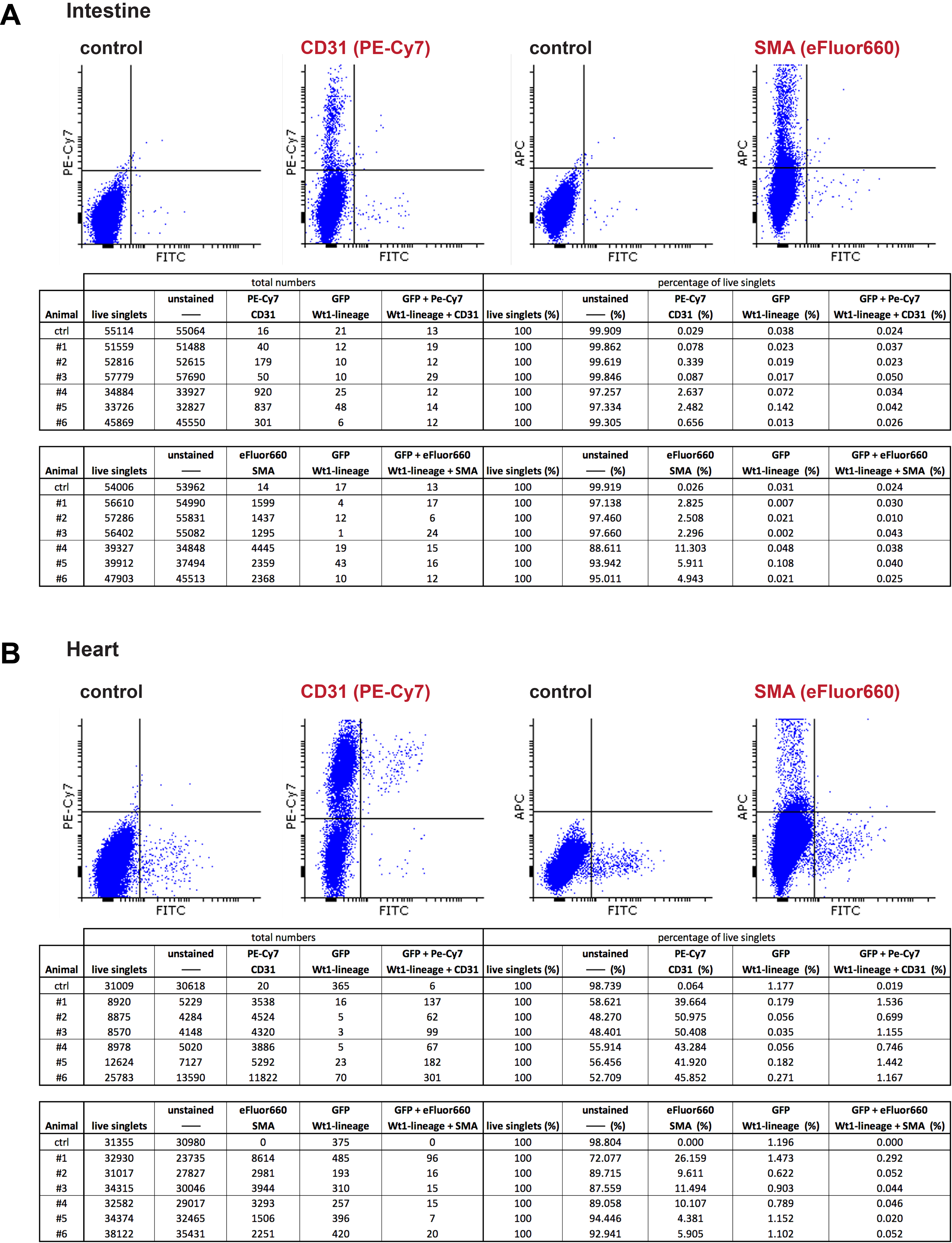


**Supplementary Figure 1**. **Flow cytometry analysis of intestine and heart from Wt1^CreERT2/+^; Rosa26^mTmG/mTmG^ mice 4 weeks and 6 months after tamoxifen administration.** A. Intestine of mice after 4 weeks (animals 1-3) and 6 months (animals 4-6) pulse-chase was dissociated and labelled for CD31 (PE-Cy7) or SMA (eFluor660). Cells were analysed after gating for live cells and singlets. Because the dissociation protocol targeted deeper tissues, relatively few GFP+ cells are present. Only very low percentages of GFP+ CD31+ or GFP+ SMA+ cells could be detected, independent of the length of the pulse-chase experiment. B. Hearts of the same mice as in (A) were labelled as described above for CD31 or SMA and analysed following the same gating strategy. About 1.2 % of all live singlet cells co-expressed GFP and CD31, while less than 0.3 % of all live singlet cells co-expressed GFP and SMA, independent of the pulse-chase duration.


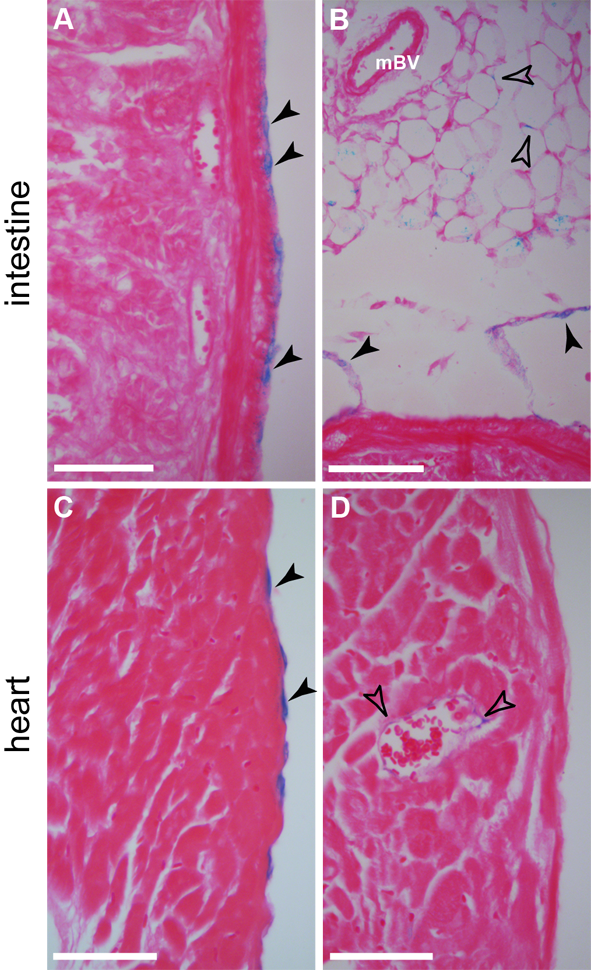


**Supplementary Figure 2: Histological analysis of mesothelial contribution to adult intestine and heart after lineage tracing of Wt1-expressing cells.** Adult Wt1^CreERT2/+^; Rosa26^LacZ/LacZ^ mice were analysed 2-4 weeks after tamoxifen administration. A, B. LacZ-positive cells in eosin counterstained paraffin sections of intestine (A) and mesentery (B) were detected in the serosal mesothelium (solid arrowheads) as well as in the mesenteric fat (hollow arrowheads); mBV, mesenteric blood vessel. Open arrowhead points towards mesenteric blood vessel. C, D. Eosin counterstained sections through ventricular wall of the heart revealed LacZ-positive cells in the epicardium (arrowheads, C) and in the coronary vessels (hollow arrowheads, F). The data shown are consistent with analyses performed in n= 5 animals. Scale bars, 50 µm (A, C, D), 100 µm (B).


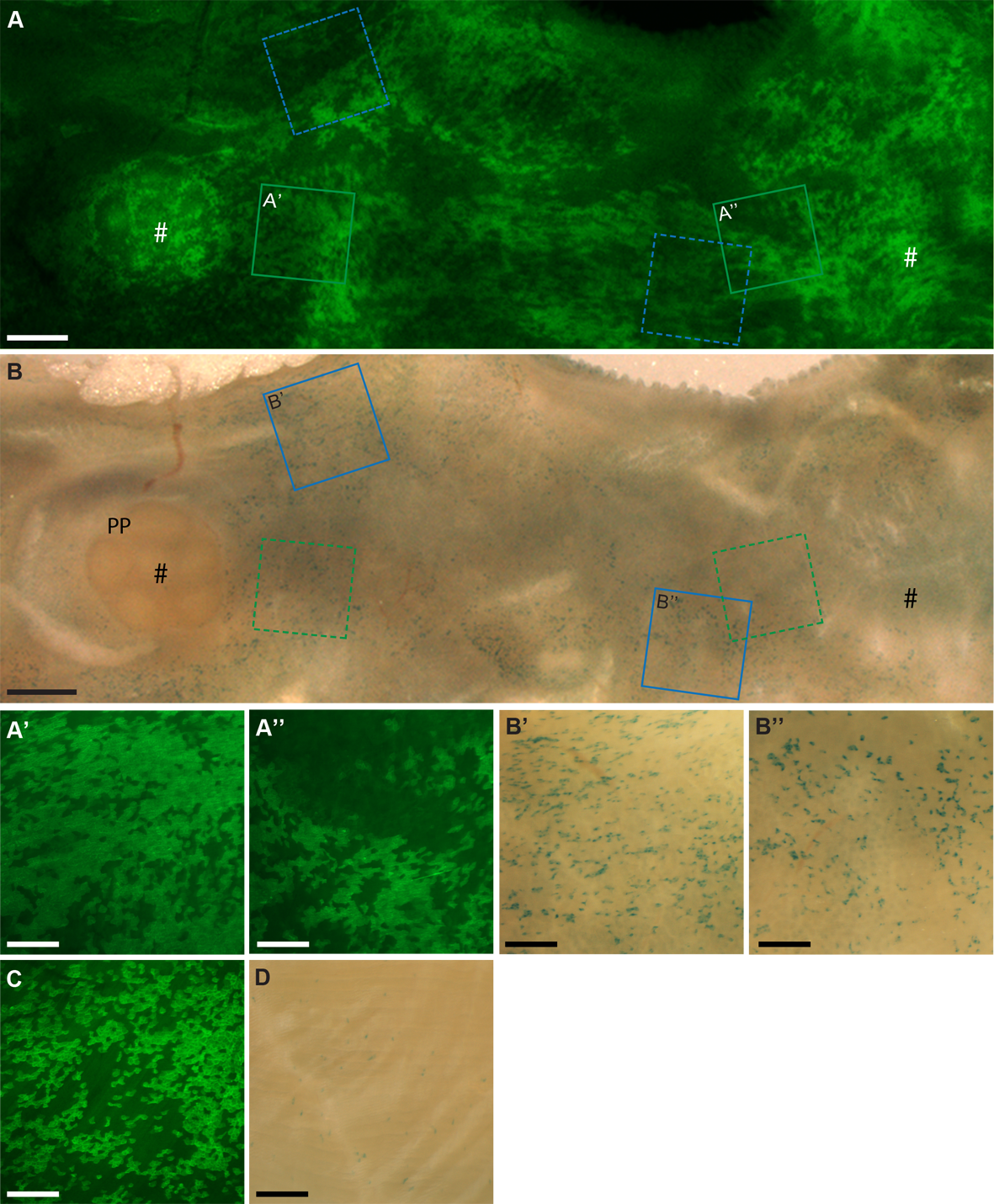


**Supplementary Figure 3: Comparison of the distribution of peritoneal GFP- and LacZ-expressing cells in the intestine and body wall muscle after lineage tracing in Wt1^CreERT2/+^; Rosa26^LacZ/mTmG^ reporter mice.** A, B. A segment of small intestine was dissected and carefully stretched out on a wax plate using fine insect pins and the profile of the GFP-expressing cells captured (A). The stretched-out tissue segment was then fixed, removed from the wax plate, XGal-stained and reattached to the same wax plate (using matching pin wholes) for capturing of the profile of LacZ-positive cells (B). In direct comparison many more GFP-positive cells overall were detected than LacZ-positive cells. Also, LacZ-free areas showed much more frequently GFP-positive cells (#) than vice versa. Specific regions of the intestinal tissue were imaged at higher magnification to demonstrate irregular coverage of GFP-expressing (A’, A’’) or LacZ-expressing cells (B’, B’’; highlighted in A and B, respectively). C, D. Coverage of GFP-positive cells in the parietal peritoneum (of the body wall muscle layer; same animal) was similarly dense while sparse for LacZ-positive cells. The data shown are consistent with analyses performed in n = 2 animals. Peyer’s patch (PP); scale bars, 1mm (A, B), 300 µm (A’, A’’, B’, B’’, C, D).


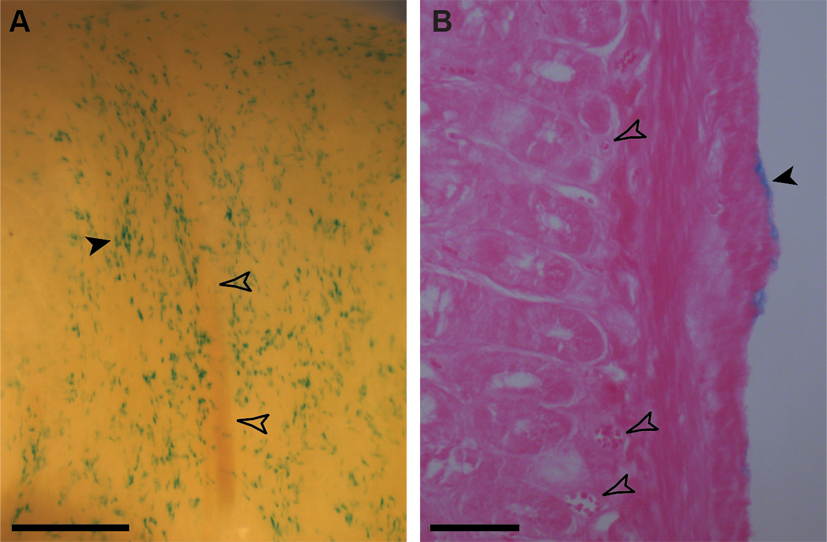


**Supplementary Figure 4: Long-term lineage tracing of Wt1-expressing cells in adult mouse intestine.** Adult Wt1^CreERT2/+^; Rosa26^LacZ/LacZ^ mice were analysed 2 and 6 months after tamoxifen administration (n = 6 in both groups). A. Whole mount analysis revealed no difference in distribution pattern and patchiness of LacZ expressing cells in the serosal mesothelium in either group (long chase shown only; filled arrowhead pointing to group of neighbouring cells, open arrowheads to blood vessel). B. Eosin counterstained paraffin sections of the same intestinal segment as shown in (A), demonstrated LacZ expressing cells in the mesothelium overlying the muscularis of the intestinal wall (filled arrowhead), but no other tissues including the vasculature (open arrowheads). Scale bars, 400 µm (A) and 50 µm (B).

**
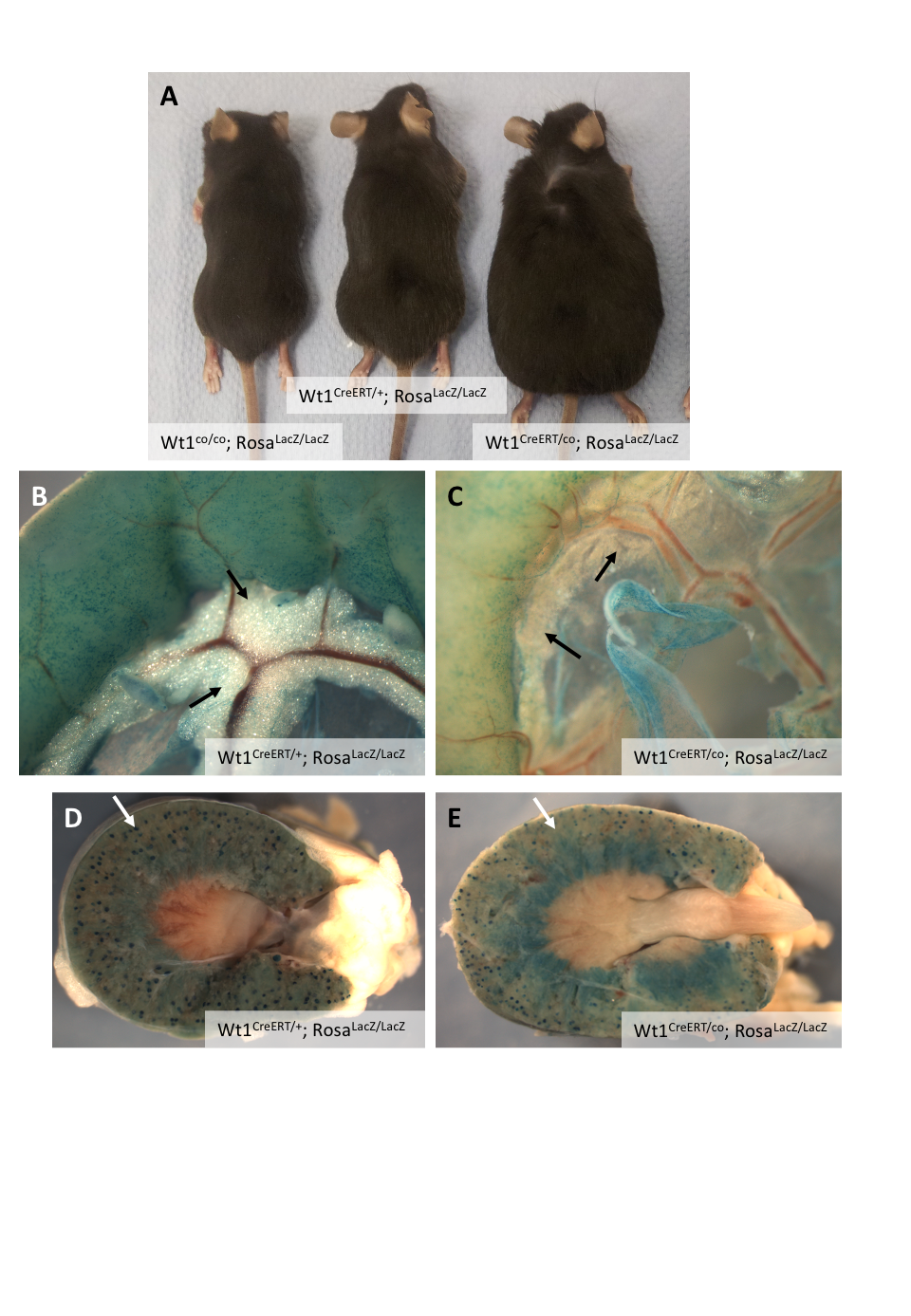
Supplementary Figure 5: Schematic illustrating the different experimental designs for this study.** Adult mice were dosed with tamoxifen on 5 consecutive days. A. In each Wt1 ablation experiment we included control genotypes for comparison (total of 10 experiments, with 2-3 Wt1^CreERT/co^; Rosa^reporter^ mice each). Only mice with the Wt1^CreERT/co^; Rosa^reporter^ genotype but not controls including mice carrying Wt1^co/co^ or Wt1^CreERT/+^, showed distention of the abdomen on day 10. B, C. Small intestine of Wt1^CreERT/+^; Rosa^LacZ/LacZ^ (B) and Wt1^CreERT/co^; Rosa^LacZ/LacZ^ mice (C) with dramatic loss of the mesenteric fat (arrows) in the Wt1^CreERT/co^; Rosa^LacZ/LacZ^ mice, while XGal staining of the mesenteric and intestinal mesothelium was not affected. D, E. Kidneys of Wt1^CreERT/+^; Rosa^LacZ/LacZ^ (B) and Wt1^CreERT/co^; Rosa^LacZ/LacZ^ mice sectioned in half revealed an apparent loss and disorganisation (arrow) of XGal-stained glomeruli in the Wt1^CreERT/+^; Rosa^LacZ/LacZ^ cortex region when compared to the control kidney.


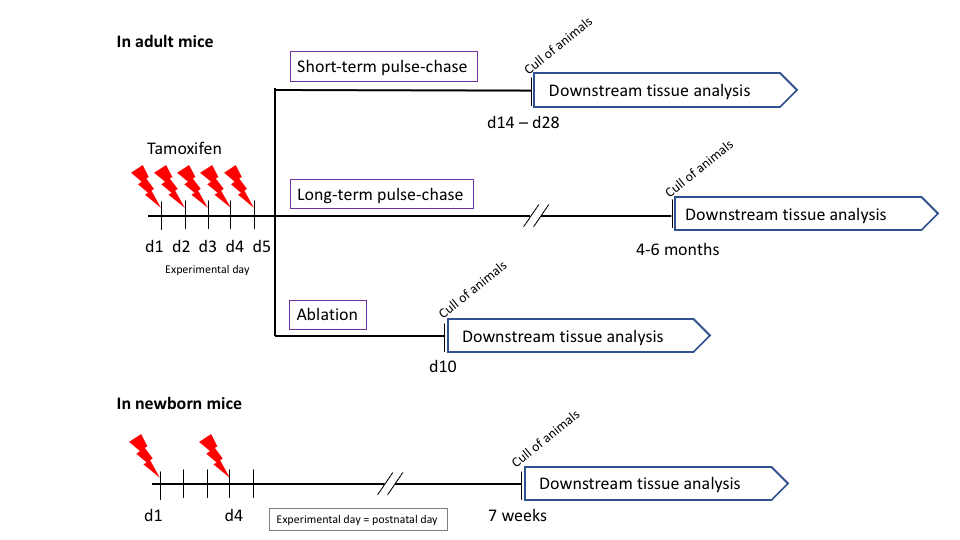


**Supplementary Figure 6: Schematic illustrating the different experimental designs for this study.** Adult mice were dosed with tamoxifen on 5 consecutive days when they have reached 8-10 weeks of age. For short-term pulse-chase, the animals were culled at between day 14 and 28 and tissues harvested for downstream analysis. For long-term pulse-chase, the tamoxifen dosing started with 5 consecutive days and the animals were culled and analysed after between 4-6 months. In the ablation experiments, animals also received tamoxifen on 5 consecutive days, followed by careful monitoring of their wellbeing from day 8 onwards. To preclude any unnecessary suffering, the mice were all culled on day 11 and tissues processed for analysis. In newborn mice, tamoxifen was administered by oral gavage to the nursing dam, on day 1 and 4 after birth, and animals culled in week 7 for downstream tissue analysis.
